# Healthcare-related transmission of mobile genetic elements co-carrying blaNDM and 16S rRNA methyltransferase genes in multiple Enterobacterales

**DOI:** 10.1099/mgen.0.001473

**Published:** 2025-08-28

**Authors:** Mark Maguire, Carlos Serna, Jose F. Delgado-Blas, Christina Clarke, Niall DeLappe, Martin Cormican, Simone C. Coughlan, Georgios Miliotis, Bruno Gonzalez-Zorn, Liam P. Burke

**Affiliations:** 1 Antimicrobial Resistance and Microbial Ecology Group, School of Medicine, University of Galway, Galway, Ireland; 2 Centre for One Health, Ryan Institute, University of Galway, Galway, Ireland; 3 Research Ireland Centre for Research Training in Genomics Data Science, Galway, Ireland; 4 Antimicrobial Resistance Unit, Animal Health Department, Faculty of Veterinary Medicine, Complutense University of Madrid, Madrid, Spain; 5 Galway Reference Laboratory Service, University Hospital Galway, Galway, Ireland

**Keywords:** 16S rRNA methyltransferases (16S-RMTase), carbapenemase, plasmids

## Abstract

Aminoglycosides are used in the treatment of serious infections with Gram-negative bacteria, especially those resistant to beta-lactams and carbapenems. 16S rRNA methyltransferases (16S-RMTase) are capable of conferring resistance to nearly all aminoglycosides. They are sometimes detected in combination with *bla*_NDM_. This study describes the mobile genetic elements associated with *bla*_NDM_ and 16S-RMTase (co-)carriage in *Enterobacterales* from Ireland in the period 2019–2023. All isolates (*n*=58) carrying both *bla*_NDM_ and a 16S-RMTase gene between 2019 and 2023 were obtained from the CPE National Reference Laboratory Service. Short-read sequences were generated for all isolates, and long-read sequences were generated for a subset of isolates (*n*=27). MOB-recon was used to distinguish plasmid-derived contigs from draft assemblies. The containment distance and DCJ-indel distance were used to find clusters of related plasmids. Isolates carrying *bla*_NDM-1_ were associated with *armA* (*n*=31) but also *rmtC* (*n*=6) carriage. These genes were co-localized most frequently on IncFIB/HI1B (*n*=12), IncM2 (*n*=10) and IncC (*n*=8) plasmids. Closely related plasmids were identified in multiple species (range: 2–5) and at different sites around Ireland; however, the IncM2 plasmids were largely associated with a single hospital. Isolates carrying *bla*_NDM-5_ were associated with *rmtB1* (*n*=28) carriage. The majority (*n*=15) were carried on a diverse range of mosaic IncF-type plasmids. Two discrete clusters of IncM1 (*n*=3) and IncFII (*n*=4) type plasmids were also detected. The study highlights the diverse plasmids co-carrying carbapenem and aminoglycoside resistance genes in Ireland. Detection of plasmids across multiple species and hospitals suggests dissemination driven by antimicrobial selective pressure and environmental reservoirs within healthcare networks. The co-dissemination of these genes on highly mobile plasmids poses a significant public health concern and emphasizes the need for greater awareness that chains of transmission of antimicrobial resistance in the healthcare setting may involve multiple species.

**Impact Statement**

This study provides a comprehensive genomic analysis of *Enterobacterales* isolates from Ireland co-harbouring *bla*_NDM_ and 16S rRNA methyltransferase (16S-RMTase) genes, which together confer resistance to both carbapenems and aminoglycosides – two critically important classes of antibiotics. By integrating short- and long-read sequencing, we characterized the mobile genetic elements responsible for the co-dissemination of these resistance determinants between 2019 and 2023. Our findings reveal a diverse array of plasmids, including IncM, IncC and mosaic IncF types, frequently shared across species and healthcare facilities, indicating ongoing horizontal gene transfer. Notably, some plasmid types, such as IncM2, exhibited localized spread, suggesting environmental reservoirs. These results underscore the complex, multispecies dynamics of antimicrobial resistance (AMR) transmission in healthcare settings and highlight the urgent need for enhanced genomic surveillance of plasmid-mediated AMR to inform infection control and stewardship strategies.

## Data Summary

All the sequence data generated in this study are available on the NCBI BioProject database under accession number PRJNA1219142 plus sample number SAMN4056362. Individual accession numbers are in Table S7 in the supplementary data.

## Introduction

Globally, beta-lactam antibiotics are extensively used in the treatment of bacterial infections [1,2]. There are four major classes: penicillins, cephalosporins, carbapenems and monobactams. Carbapenems are commonly used as a drug of ‘last resort’ for the treatment of extensively resistant *Enterobacterales*. Carbapenemase-producing *Enterobacterales* (CPE) display reduced susceptibility to carbapenems due to the production of acquired beta-lactamases, the most important of which are *bla*_KPC_, *bla*_OXA_, *bla*_NDM_, *bla*_VIM_ and *bla*_IMP_ types [3].

Since the first CPE were identified in 1993, they have expanded and disseminated globally [4,5]. As a result, since 2017, the World Health Organization has designated CPE as a priority pathogen, listed among the most urgent antimicrobial-resistant threats [6]. In Ireland, the number of CPE isolates referred to the National CPE Reference Laboratory service increased by 117% between 2019 and 2024. While *bla*_OXA_ is the most commonly detected type of carbapenemase in Ireland to date, *bla*_NDM_ detection has increased by 132% between 2019 and 2024, and it is now the second most detected carbapenemase [7]. Dissemination of metallo-carbapenemases such as *bla*_NDM_ poses a particular problem, as therapeutic options for resistance mediated by these enzymes are more limited than for other carbapenemases. These carbapenemase genes (CGs) are often associated with a range of mobile genetic elements (MGEs), such as plasmids and transposons. These MGEs can facilitate their spread both within and between numerous bacterial species and genera [8]. The MGEs can often carry numerous other antimicrobial resistance (AMR) determinants to antimicrobials used in the treatment of carbapenem-resistant organisms, resulting in multidrug-resistant organisms.

Aminoglycosides are broad-spectrum antimicrobials that function by inhibiting protein synthesis in bacteria. They are often used in combination with other antimicrobials and are useful in the treatment of infections with CPE that remain susceptible to these agents [9]. Aminoglycoside resistance can occur through several different mechanisms: (i) enzymatic modification and inactivation of the antibiotic, (ii) increased efflux, (iii) decreased permeability and (iv) target site modification [10]. Aminoglycoside modifying enzymes are the most common aminoglycoside resistance mechanism in *Enterobacterales*, with an ever-growing number of enzymatic variants capable of utilizing various aminoglycoside antimicrobials as substrates [11]. However, target site modification, through the production of 16S rRNA methyltransferases (16S-RMTases), is of particular concern clinically. 16S-RMTases modify the A site of 16S rRNA, preventing the antimicrobial from interacting with its target and resulting in high-level resistance to all clinically relevant aminoglycosides. To date, there are 12 16S-RMTases (*armA*, *rmtA-I* and *npmA-B*), mostly detected in Gram-negative bacteria; a*rmA* and *rmtB* have been detected globally and are usually carried on MGEs, contributing to their rapid dissemination [12].

In 2017, CPE were declared a public health emergency in Ireland, and since then significant effort has been made to identify and control the spread of CPE [13]. Most patients admitted into acute hospitals in Ireland are offered screening for CPE carriage through the collection of a rectal swab or stool sample. CPE are detected through the use of selective culture, enzyme detection or molecular testing. The first patient isolate of each species of CPE is referred to the CPE National Reference Laboratory Service (CPERL). Whole-genome sequencing (WGS) is carried out on all isolates, and bioinformatic analysis identifies the resistance genotype and certain carbapenemase-carrying plasmids. Core genome multilocus sequence typing is used to identify potentially related isolates. This approach does not identify the spread of resistance genes related to horizontal gene transfer [14].

In this study, we investigate the molecular epidemiology of isolates co-carrying *bla*_NDM_ and 16S-RMTase collected in Ireland between 2019 and 2023 and characterize their associated plasmids and transmission dynamics. Using both second- and third-generation sequencing, we assess the role of MGEs in the spread of these genes in the Irish healthcare system and environment.

## Methods

### Isolate selection

We searched public databases and the CPERL database for *Enterobacterales* isolated in Ireland from any source (including human, animal and environment) between 2019 and 2023 that co-carried *bla*_NDM_ with at least one 16S-RMTase gene. A total of 58 isolates were included in the study and comprised all 56 recoverable isolates with this resistance gene pattern submitted to the CPERL by hospitals in Ireland and a further two environmental isolates collected from airport sewage in 2019 and a river in 2020 within the Antimicrobial Resistance and the Environment – Sources, Persistence, Transmission and Risk Management (AREST) research study [15].

The 56 CPERL isolates co-carried *bla*_NDM-1_ (*n*=34) or *bla*_NDM-5_ (*n*=22) with at least one 16S-RMTase gene. They were collected from 20 healthcare facilities in Ireland from a wide variety of sample types: environmental, including hospital sinks (*n*=2) and hospital toilets (*n*=1); human, including faeces/rectal screening swabs (*n*=40), urine (*n*=10), sputum (*n*=1) and wound swabs (*n*=2). CGs were initially detected in each hospital by molecular (PCR) or biochemical (CARBA-NP test, NG-Test CARBA 5) means and subsequently confirmed by the CPERL via WGS. Patient metadata followed data minimization principles to protect patient identity.

### Antimicrobial susceptibility testing

The minimum inhibitory concentration (MIC) of meropenem, gentamicin and amikacin was determined using gradient strips (Liofilchem). MIC was controlled using *Escherichia coli* ATCC 25922 and *Pseudomonas aeruginosa* ATCC^®^ 27853. Susceptibility to meropenem (*S*≤2 mg l^−1^, *R*≥8 mg l^−1^), gentamicin (*S*≤2 mg l^−1^, *R*>2 mg l^−1^) and amikacin (*S*≤8 mg l^−1^, *R*>8 mg l^−1^) was interpreted according to EUCAST guidelines version 14.0 [16].

### DNA extraction and sequencing

All 58 study isolates that carried both *bla*_NDM_ and a 16S-RMTase gene, including 56 recoverable hospital isolates and the 2 AREST environmental isolates, were sequenced upon receipt at CPERL. DNA was extracted from these isolates using the EZ1 advanced XL machine and the EZ1 DNA tissue kit (Qiagen). This DNA was then used to prepare a library using the Nextera DNA Prep Library Preparation Kit (Illumina, San Diego, CA, USA). This library was sequenced using the MiSeq platform (Illumina, USA), generating PE300 reads.

A subset of isolates (*n*=27) was selected for long-read sequencing based on diversity of species, sequence types, geographical regions and resistance/plasmid profiles. All isolates that were producing either two CGs (*n*=8) or two 16S-RMTase genes (*n*=4) were selected. For isolates belonging to the same sequence type (ST), only one representative was chosen, unless an isolate from a site of infection (e.g. urine and sputum) was available, in which case it was also included. All healthcare environment isolates (*n*=5) were selected for sequencing. The remainder (*n*=10) were selected randomly to cover as diverse a collection of species and locations as possible.

Long-read sequencing was performed using the Oxford Nanopore MinION FLO-MIN114 R10 flow cell (Oxford Nanopore Technologies, UK). DNA extraction was carried out using the GeneJet Genomic DNA Purification kit (Thermo Fisher Scientific Baltics, Lithuania). The extracted DNA was quantified on the Qubit (Invitrogen Corp., Carlsbad, CA, USA) fluorometer and stored at −20 °C. The library was prepared using the SQK-RBK114 rapid barcoding kit (v14) (Oxford Nanopore Technologies, Oxford, UK). The library was loaded into the flow cell and run on the MinION Mk1c for 72 h. Basecalling of the raw Fast5 files was carried out onboard using the MinKNOW software (v 22.12.5) using the Fast basecalling model.

### *De novo* assembly and genetic content analysis

Quality control, quality filtering and adapter trimming of short-read sequences were carried out using fastp (v0.23.2) [17]. Reads with a Phred quality score of less than Q15 and length less than 15 bp were filtered out using default parameters. Sequence quality control and filtering of long reads were carried out using filtlong (v0.2.1) (https://github.com/rrwick/Filtlong) using default settings, with extensions --min_length 1,000 and –keep_percent 95. Hybrid genome assembly was carried out using Unicycler (v0.5.0) [18] with default parameters and no post-assembly polishing. Short-read-only assembly was carried out using shovill (v1.1.0) (https://github.com/tseemann/shovill). The quality of assemblies was assessed using QUAST (v5.2.0).

The AMR profile of the assembled genomes was assessed using ABRicate (v1.0.1) (Seemann T, 2019 Abricate, Github https://github.com/tseemann/abricate) using the NCBI AMRFinderPlus [19], PlasmidFinder [20] and virulence factor [21] databases to determine the resistance genes, plasmid content and virulence genes, respectively. ABRicate was run using default settings of 80% identity and coverage.

Hybrid genome assemblies were visualized using Bandage (v0.81) [22], and chromosomal and plasmid contigs were sorted into separate FASTA files. Only closed, circular plasmids were used for downstream analysis.

Annotation of chromosomal and plasmid contigs was carried out using Bakta (v1.4.0) to determine the complete gene content and gene families. Annotation was via a large taxonomy-independent database (downloaded: 8 April 2022) using UniProt’s entire UniRef protein sequence cluster universe [23].

### Plasmid content and structure analysis

The MOB-suite tool MOB-typer was used to predict the replicon family, relaxase type, mate-pair formation type and predicted transferability of the plasmids obtained from the hybrid sequenced isolates. The MOB-recon tool was used to reconstruct plasmid sequences from draft assemblies using the clustered plasmid reference database provided by MOB-cluster [24]. Plasmid multilocus sequence typing (pMLST) was used to identify the sequence types associated with the plasmids where an appropriate pMLST scheme was available [20].

Clusters of related plasmids were identified using Pling [25] on closed, circular plasmids; the default containment distance of <0.5 was used for inclusion in the cluster. Networks of related plasmids were generated, displaying the containment distance and double cut and join indel (DCJ-indel) distance (Fig. S1, available in the online Supplementary Material). Plasmid percentage coverage and identity were compared for all plasmids in each cluster (see Table S6), and closely related plasmids were mapped using Proksee [26]. Plasmid incompatibility type, AMR gene content and blast comparison (https://blast.ncbi.nlm.nih.gov/Blast.cgi) were used to match predicted plasmids to closed plasmid clusters. Predicted plasmids that had >60% coverage of a closed plasmid were mapped and visualized using Proksee.

## Results

A total of 58 isolates were analysed, carrying combinations of *bla*_NDM_ variants and 16S-RMTase genes. These isolates were collected from 20 healthcare facilities (*n*=56), a river water sample (*n*=1) and an airport sewage sample (*n*=1). Closed, circular plasmids were obtained for 27 isolates, while the remainder (*n*=31) had plasmids predicted from short-read assemblies.

The majority of *bla*_NDM-1_ isolates were associated with *armA* (*n*=28), comprising those with *armA* alone (*n*=24) or in combination with a second 16S-RMTase: *rmtF* (*n*=3) or *rmtC* (*n*=1). Six *bla*_NDM-1_ isolates carried *rmtC* alone. A single isolate carried *bla*_NDM-1_ with *rmtF*. Carriage of *bla*_NDM-5_ was almost exclusively linked to *rmtB1* (*n*=23), with two of these isolates also carrying *armA*. Where closed plasmids were obtained, most *bla*_NDM_/16S-RMTase genes were plasmid-borne and located on the same plasmid (25/27), with IncF and IncM plasmids dominating. In a single isolate, the genes were located on the chromosome, and in another, they were located on separate plasmids. Below, we describe the characteristics of these plasmids and their potential transmission routes.

Of the 58 isolates in the study, 56 were successfully cultured from frozen bead stocks. Regarding AMR phenotypic profiles, all (*n*=56) displayed high-level resistance to the aminoglycosides gentamicin and amikacin, while 46/56 isolates were resistant to meropenem. Briefly, 5/56 isolates were intermediate to meropenem, and 5/56 were susceptible to meropenem (see Table S5). As these susceptible phenotypes were unexpected, the meropenem MIC assays were repeated, and PCR was used to confirm the presence of *bla*_NDM_ in the tested isolates. No SNPs were detected in *bla*_NDM_ or the associated promoter sequences that might explain the phenotypes. NDM-producing *Enterobacterales* with meropenem MICs within the susceptibility range have been reported previously, including by the CPERL in Ireland [27].

### Co-carriage of *bla*_NDM-1_ and *armA* primarily associated with two discrete plasmid clusters

A total of 28 isolates spanning seven species: *Klebsiella pneumoniae* (*n*=14), *Klebsiella michiganensis* (*n*=2), *E. coli* (*n*=7), *Enterobacter chengduensis* (*n*=2), *Providencia stuartii* (*n*=1), *Citrobacter amalonaticus* (*n*=1) and *Leclercia adecarboxylata* (*n*=1) were identified as carrying both *bla*_NDM-1_ and *armA*. Two of the *K. pneumoniae* isolates also carried an additional CG, *bla*_OXA-48_. Also, some isolates carried additional 16S-RMTase genes, including one *P. stuartii* which co-carried *rmtC* (*n*=1) and three *K. pneumoniae* which co-carried *rmtF1*. These isolates were recovered from 13 healthcare facilities and a river water sample.

Closed plasmids were obtained for nine isolates, and in eight of these, the *bla*_NDM-1_ and *armA* were detected on the same plasmid. In the other case, the genes were located on separate plasmids. In a single case, the plasmid carrying *bla*_NDM-1_ and *armA* was also carrying *bla*_OXA-48_ (Table S2).

Two main clusters of plasmids carrying *bla*_NDM-1_ and *armA* were identified: a diverse IncF plasmid disseminating across species and locations and an identical plasmid persisting in a single healthcare facility.

**Fig. 1.**
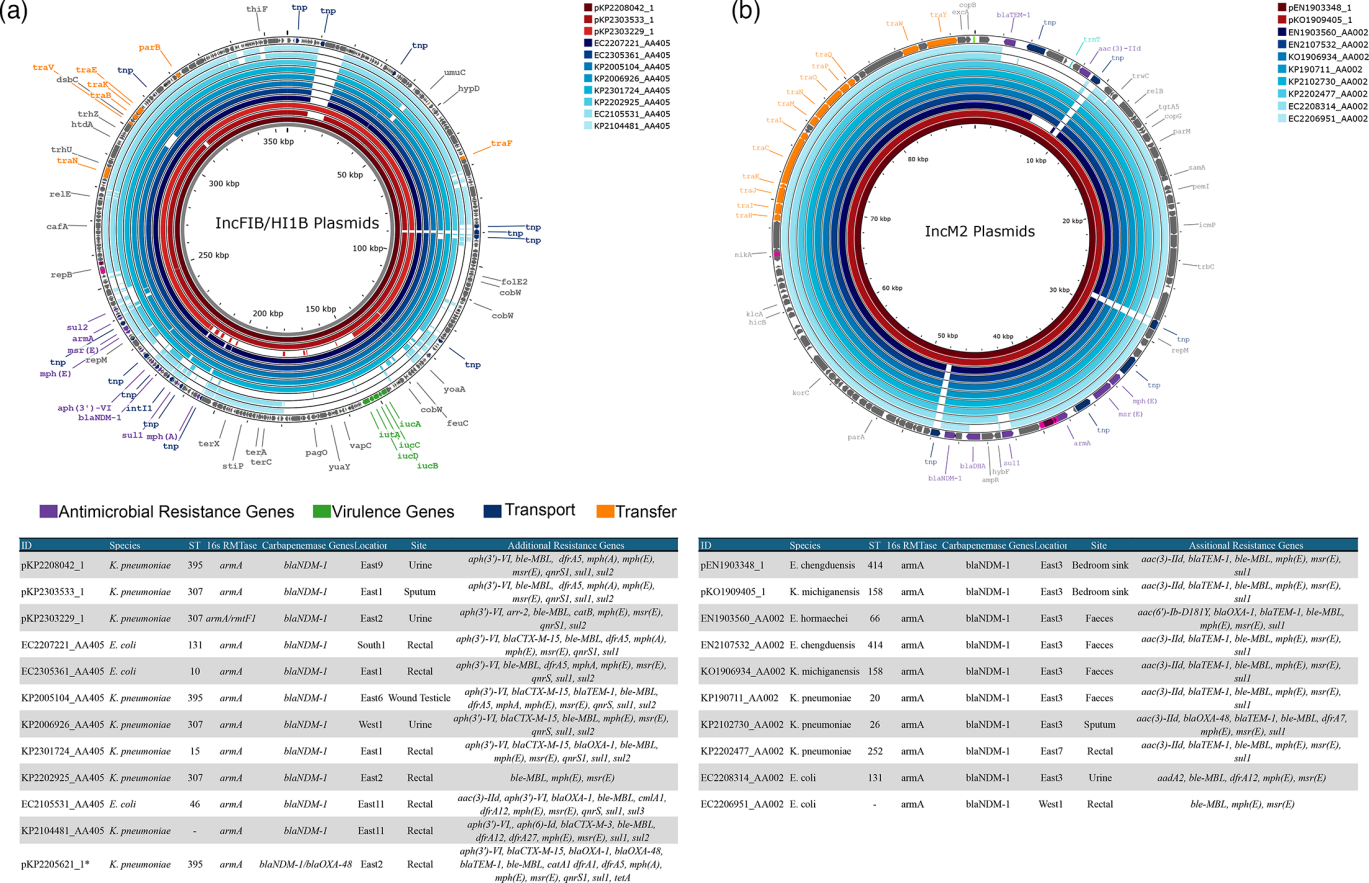
Comparison of plasmids carrying *bla*_NDM-1_ in conjunction with *armA*. Inner rings in red represent closed circular plasmids. Outer rings in blue represent plasmids predicted from short-read assemblies. Plasmids are mapped to the innermost ring, which was used as a reference.

Cluster 1: IncFIB(pNDM-Mar)/IncHI1B(pNDM-MAR) plasmid (~299–436kb) detected in four *K*. *pneumoniae* isolates (ST395, ST307) from four hospitals in the East of Ireland. These plasmids also harboured genes (*rmpA2*, *iutA*, *iucABCD*) associated with hypervirulence. Based on the prediction of plasmids from short-read assemblies using MOB-recon, an additional eight isolates carried plasmids similar to this cluster. These were predicted in *K. pneumoniae* ST395/ST307 (*n*=5) but also *E. coli* (*n*=3) (Fig. 1a).

This plasmid was almost identical to previously reported plasmids detected in *K. pneumoniae* ST383 (CP034201.2), identified in the UK. A number of previously identified plasmids were also comparable to these plasmids but lacked the *bla*_NDM-1_ and surrounding region. These plasmids were all detected in *K. pneumoniae* isolates collected in the UK: ST147 (CM007852.1 and CP040726.1); India: ST2096 (MK649825.1); Russia: ST512 (PQ126483.1), ST147 (PQ126450.1), ST512 (PQ126484.1/PQ126485.1) and ST395 (PQ126471.1).

Cluster 2: two identical IncM2 plasmids (~89 kb), detected in two isolates from the same healthcare facility (*K. michiganensis* and *En. chengduensis* from a bathroom sink), with a shared AMR gene profile. Six additional short-read plasmids matched this cluster and were predominantly in isolates from the same healthcare facility as above (Fig. 1b).

These plasmids were similar to a large number of plasmids from diverse global locations, such as studies in the UK [28] and Hong Kong [29]. Some of the plasmids in those studies lacked *armA* and the surrounding region.

In a single isolate (B20351), *bla*_NDM-1_ and *armA* were carried on an IncFIB(K)(pCAV1099-114)/IncHI1B(pNDM-MAR) type plasmid. This isolate was collected from river water in the south of Ireland. This plasmid was non-mobilizable and was not a match for any of the other plasmids identified in this study.

The *armA* gene in all plasmids in this study was located in a region consisting of *armA-permease-ISEc29-msr(E)-mph(E*) flanked by two copies of IS*6*. Previous studies have reported *armA* located within a similar composite transposon Tn*1548*, which is capable of being transposed to other genomic sites [30,32].

### Co-carriage of *bla*_NDM-1_ and *rmtC* associated with a conjugative IncC plasmid

Isolates carrying both *bla*_NDM-1_ and *rmtC* (*n*=7) were from three species – *K. pneumoniae* (*n*=2), *Enterobacter hormaechei* (*n*=3) and *P. stuartii* (*n*=2). The isolates originated from five healthcare facilities. Closed genomes were generated for five of these isolates, and in all cases, *bla*_NDM-1_ and *rmtC* were co-carried on the same plasmid. In one case, the plasmid also carried *armA*.

**Fig. 2.**
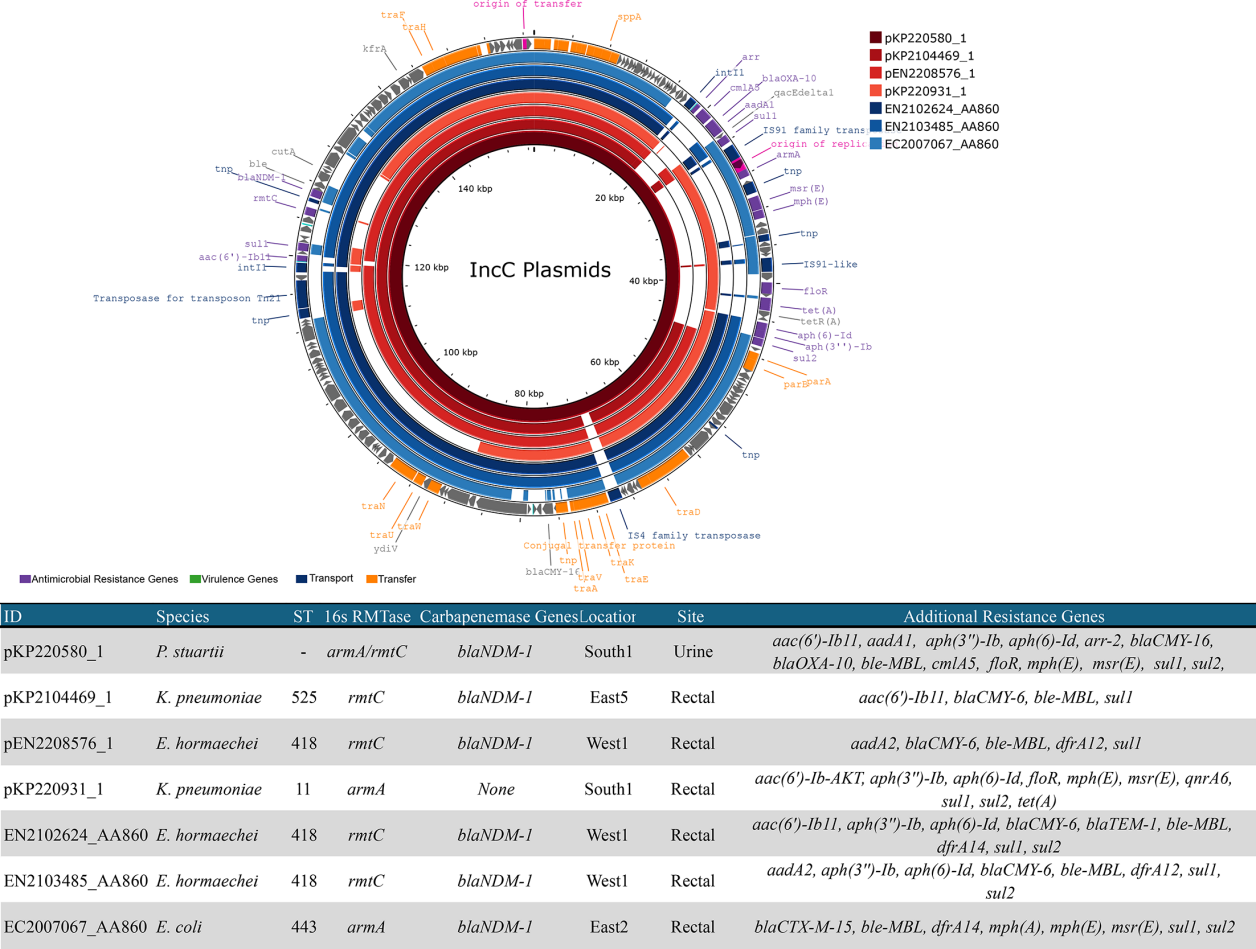
Comparison of IncC-type plasmids. Each ring represents a single plasmid. Inner rings in red represent closed circular plasmids. Outer rings in blue represent plasmids predicted from short-read assemblies. Plasmids are mapped to the innermost ring, which was used as a reference.

A single plasmid cluster associated with these isolates was detected. This IncC-type plasmid was identified in three isolates of three different species and varied in length from 132 to 157 kb. In *K. pneumoniae* and *En. hormaechei*, this plasmid carried *bla*_NDM-1_ alongside *rmtC* (pKP2104469_p1 and pEN2208576_p1, respectively), whereas in *P. stuartii*, the plasmid carried *bla*_NDM-1_ with *rmtC* and *armA* (pKP220580_p1). It was identified in three healthcare facilities in distinct regions between 2021 and 2022. A plasmid in a *K. pneumoniae* isolate carrying *armA* alone (pKP220931_p1) from the same facility as pKP220580_p1 was also a match for this cluster. Three additional isolates with similar plasmids were predicted to be part of this cluster. These plasmids all carried *bla*_NDM-1_ in combination with *rmtC* (*n*=2, *En. hormaechei*) and *armA* (*n*=1, *E. coli*) (see Fig. 2).

The backbone of these plasmids was a match for a large number of other plasmids carrying *bla*_NDM-1_ and *rmtC* in Hong Kong (CP050164.1, KP) and Australia (CP015835.1, EC) or *armA* only in Denmark (MG450360.1, EC) and Sweden (CP030720.1, KP). No good match for plasmid pKP220580_p1, carrying both resistance islands for *bla*_NDM-1_/*rmtC* and *armA*, was identified in GenBank (21 January 2025).

In two other isolates carrying *bla*_NDM-1_ and *rmtC*, the genes were located on an IncC-type plasmid unrelated to the above cluster (pKP2202517_p1) and an IncFIB/FII/FII-type plasmid (pKP2006286_p1) (Table S2).

Although all plasmids within the IncC plasmid cluster had a similar genetic environment surrounding *rmtC* and *bla*_NDM-1_, the genetic context varied considerably for the other plasmids carrying *rmtC*. While plasmid pKP2006286_1 exhibited a similar region downstream of the genes, the upstream region displayed no structural similarity. In plasmid pKP2202517_1, *rmtC* and *bla*_NDM-1_ were harboured within a genetic context that was entirely distinct from that of the other plasmids analysed.

### Isolates carrying *bla*_NDM-1_ with *rmtF1* alone

A single *K. pneumoniae* isolate (KP1908981) carried *bla*_NDM-1_ in combination with *rmtF1*. These genes were located on an IncFIB(K)/IncFII(pKP91)-type plasmid (187 kb). This plasmid carried nine additional resistance genes: *aac(6′)-Ib-G*, *aadA2*, *aph(3′)-Ia*, *arr-2*, *ble_MLB_*, *catB*, *dfrA12*, *mph(A*) and *sul1*. No other plasmids co-carrying *bla*_NDM-1_ and *rmtF* were identified in publicly available databases (NCBI/plsdb).

### Co-carriage of *bla*_NDM-5_ with *rmtB1* is associated with diverse plasmids

Isolates carrying both *bla*_NDM-5_ and *rmtB1* (*n*=28) were from seven different species – *E. coli* (*n*=18), *K. pneumoniae* (*n*=4), * En. hormaechei* (*n*=2), *Citrobacter braakii* (*n*=1), *Citrobacter freundii* (*n*=1), *Klebsiella oxytoca* (*n*=1) and *Raoultella ornithinolytica* (*n*=1). Seven of these carried second CG – *bla*_OXA-181_ (*n*=4), *bla*_OXA-232_ (*n*=1), *bla*_OXA-244_ (*n*=1) and *bla*_OXA-48_ (*n*=1). Two isolates carried *armA* also.

Closed genomes were generated for 13 of these isolates. In 12 isolates, the *bla*_NDM-5_ and *rmtB1* genes were located on the same plasmid. In one of these plasmids, *armA* was also located on the same plasmid. In the remaining isolate, an *E. coli* ST38, *bla*_NDM-5_ and *rmtB1* were all detected on the chromosome.

**Fig. 3.**
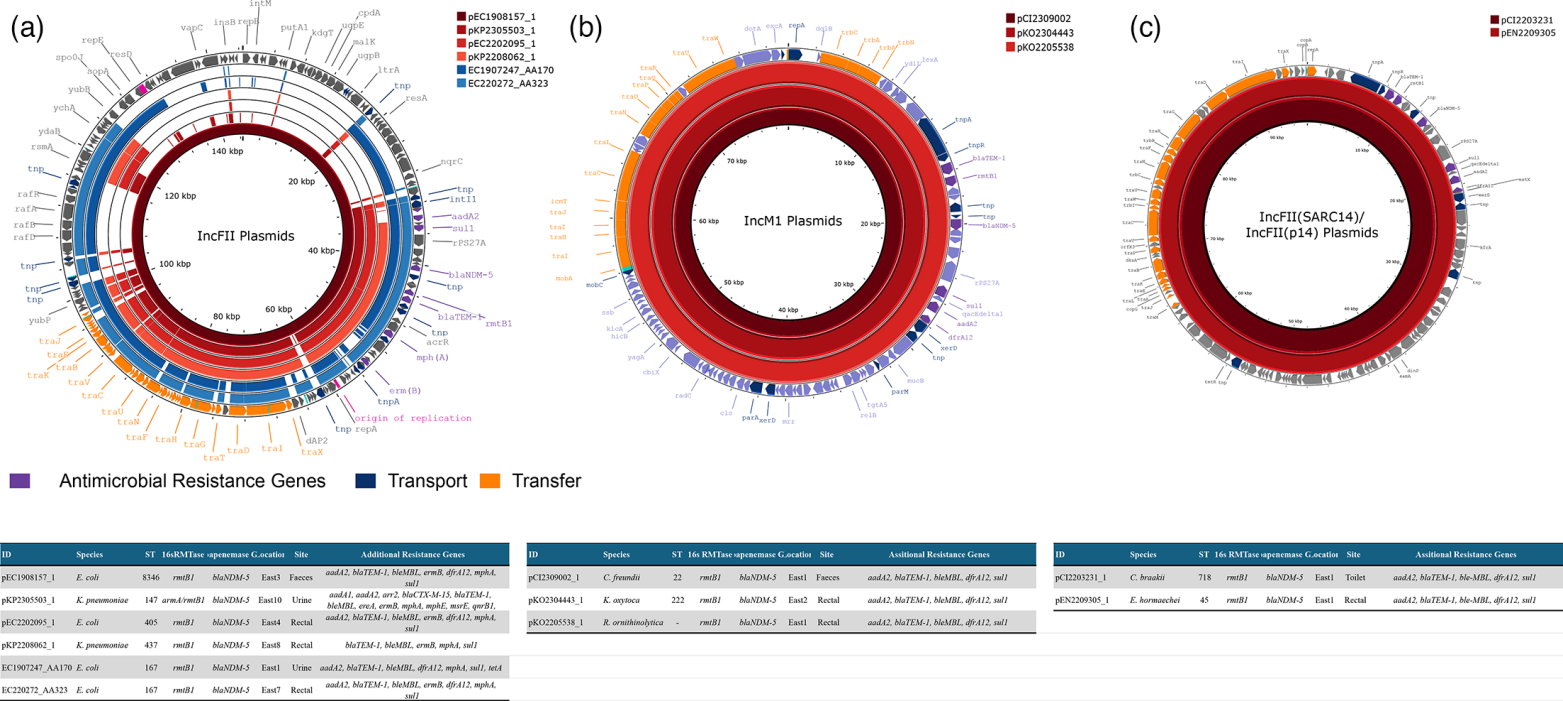
Comparison of plasmids carrying *bla*_NDM-5_ and *rmtB1*. Inner rings in red represent closed circular plasmids. Outer rings in blue represent plasmids predicted from short-read assemblies. Plasmids are mapped to the innermost ring, which was used as a reference.

The following three clusters of plasmids were identified in these isolates (Fig. 3):

Cluster 1, a variable IncF-type plasmid (~94–144 kb), was identified in four isolates [*K. pneumoniae* (*n*=2) and *E. coli* (*n*=2)] from four healthcare facilities in the East of Ireland. One of these also carried *armA* on the same plasmid. An additional two predicted plasmids matched this cluster. These were both identified in *E. coli* ST167 from two distinct healthcare facilities in the East of Ireland (Fig. 3a). These plasmids were a close match for those described by Turton *et al*. [33] in the UK.

Cluster 2, an identical IncM1-type plasmid (~80 kb) in three species – *C. freundii*, *K. oxytoca* and *R. ornithinolytica*. These isolates were collected from two healthcare facilities in the East of Ireland within a 1-year period. No predicted plasmids were a good match for this cluster, and while a number of plasmids with a similar backbone were detected, no other plasmids carrying both *bla*_NDM-5_ and *rmtB1* were discovered (see Fig. 3b).

Cluster 3, an identical IncFII(SARC14)/FII(p14) plasmid (~96 kb) from two different species, was collected from a rectal screen swab from a patient (*En. hormaechei*) and a hospital toilet (*C. braakii*) in the same location over a 14-month period. No predicted plasmids were a match for this cluster (see Fig. 3c).

Two closed genomes carrying *bla*_NDM-5_ and *rmtB1* did not match any other isolates from this study. In isolate B19695, *bla*_NDM_ and *rmtB1* were located on the chromosome. This isolate was collected from airport sewage in the East of Ireland. In isolate EC2208985, the genes were carried on a ColKP3/IncX3-type plasmid (~74 kb). This plasmid also carried *bla*_OXA-181_.

The genetic environment surrounding *rmtB1* was the same in isolates from this study. This region consisted of *tnpR-bla*_TEM-1_-*rmtB1*-Na(+)/H(+) antiporter-chaperonin-IS*6* and was the same when carried on a plasmid or the chromosome. This region is similar to that commonly reported with *rmtB* located downstream of a Tn*3*-like transposon and *bla*_TEM_ and a variable region downstream of *rmtB* [31,34].

## Discussion

This study characterized a diverse collection of *Enterobacterales* isolates co-carrying *bla*_NDM_ and 16S-RMTases. While some evidence of clonal expansion was observed, no dominant species or STs were observed either within individual hospitals or at a national level. *K. pneumoniae* and *E. coli* were the most common species but may be overrepresented due to the clinical nature of the dataset used. Strong associations between *bla*_NDM-1_ and *armA* and between *bla*_NDM-5_ and *rmtB1* were noted. In nearly all cases, these resistance genes were co-located on the same plasmids, which were often conjugative (as predicted by MOB-typer). These plasmids were frequently shared across multiple species, highlighting the key role of plasmid-mediated dissemination of these resistance genes.

Of particular interest is the finding of clusters of identical plasmids within a single healthcare facility, persisting over several years, such as IncM1 and IncM2 plasmids. These plasmids were detected in multiple species and over an extended period. This is consistent with the persistence and spread of these plasmids within a facility. Hospital plumbing systems, especially sinks, may act as reservoirs for CPE and the plasmids associated with the carriage of CGs. Studies examining hospital plumbing systems have found similar examples of long-term persistence of both species and plasmids associated with CGs in unrelated hosts and environmental reservoirs in the hospital setting [35,36].

Clusters of highly variable IncF- and IncC-type plasmids detected in multiple healthcare facilities were often related to plasmids from multiple other locations globally, including the UK, Europe, Hong Kong and Australia, supporting the involvement of both international travel and widespread MGE dissemination as drivers in the dissemination of AMR [37,38]. The process may be supported by amplification in healthcare settings under intense selective antimicrobial pressure, as suggested by the finding in relation to IncM1 and IncM2 plasmids (above).

Two clusters of plasmids co-carrying *bla*_NDM-1_ and *armA* were identified. The first was an IncFIB/IncH1B plasmid identified in multiple healthcare facilities. These plasmids also harboured genes associated with hypervirulence in *K. pneumoniae* [39,40]. These plasmids represent the convergence of carbapenem resistance and hypervirulence, and their acquisition, especially by * K. pneumoniae*, has the potential to create strains that can cause severe infections with limited treatment options [41]. Of note, we identified the globally disseminated high-risk pathogenic *K. pneumoniae* ST307 clone carrying this plasmid [42]. The close similarity of these IncFIB/IncHIB plasmids to those from international isolates suggests global dissemination.

The second cluster was an IncM2-type plasmid that was predominantly detected in a single healthcare facility. This plasmid was identified in both patient samples and the hospital environment over a 3-year period, suggesting an environmental reservoir within the facility. Plasmids in this cluster were similar to numerous other plasmids on NCBI carrying the *bla*_NDM-1_ gene but lacking *armA* and the surrounding region. These studies found the plasmids to have a wide geographical spread and detection in multiple bacterial taxa [28,29]. The *armA* gene was likely acquired via a transposable element [30].

A single cluster associated with *bla*_NDM-1_ and *rmtC* was identified in an IncC-type plasmid. This plasmid had a shared backbone but carried variable resistance genes located on genomically predicted mobilizable regions. IncC-type plasmids are globally distributed and commonly associated with the carriage of *bla*_NDM-1_ and *rmtC* [43,44]. The ability of these plasmids to acquire other multidrug resistance (MDR) regions demonstrates the mosaic nature and genomic flexibility of MDR plasmids.

Three clusters of plasmids co-carrying *bla*_NDM-5_ and *rmtB1* were identified. The first was a highly variable IncF-type plasmid detected in *K. pneumoniae* and *E. coli*, isolated from multiple facilities over a 4-year period. Similar to a study by Turton *et al*. in England, plasmids carrying *bla*_NDM-5_ and *rmtB* were non-identical but shared common structures and were widely distributed across multiple healthcare facilities [33].

Two clusters, IncM1- and IncFII-type plasmids, were predominantly from the same facility and consisted of identical plasmids detected in multiple species. While both of these plasmid types are associated with global spread, no significant matches carrying *bla*_NDM-5_ and *rmtB1* were found in online databases (GenBank); however, similar plasmids lacking this region were detected. The diverse array of plasmids detected carrying *armA* and *rmtB1* may be partially explained by the conserved genomic structure observed surrounding these genes. These regions were found in multiple plasmid types and shared homology with previously identified transposable regions (e.g. Tn*1548*), which have been demonstrated to be capable of mobilization [31,32, 34].

Two isolates from outside the hospital environment were also included in this study. Neither of these was a match in terms of ST or plasmid to any of the hospital isolates in this study. In these *K. pneumoniae* isolates, the *bla*_NDM_ and 16S-RMTase genes were co-carried on an IncF-type plasmid and the chromosome. No other isolates from the general environment or animals were available for analysis.

Our findings are limited by incomplete epidemiological data, restricting precise identification of transmission pathways, an area recommended for future research. Furthermore, long-read sequencing was not available for all isolates. Closed circular plasmids were only available for 27 isolates. The remaining plasmids were predicted from short reads and were not circular or complete and may not fully represent the actual plasmid.

Future work should focus on the validation of plasmid and transposon mobility through functional assays to confirm their role in the spread of these resistance genes. Long-read sequencing should be employed to enhance the reconstruction of relevant bacterial genomes and enable a more comprehensive understanding of their structure and mobile elements. Enhanced environmental sampling, particularly in hospitals with high numbers of CPE and 16S-RMTase producers, could enhance our understanding of environmental reservoirs in the healthcare setting and how they are contributing to the spread of AMR.

## Conclusion

This study highlights that diverse plasmids carrying *bla*_NDM_ and 16S-RMTase readily spread in healthcare networks within geographically connected settings. Surveillance should therefore target MGEs across species and hospitals to uncover hidden transmission pathways.

## Supplementary material

10.1099/mgen.0.001473 Uncited Table S1.

10.1099/mgen.0.001473 Uncited Fig. S1.

## Abbreviations

AMR: antimicrobial resistance
AREST: Antimicrobial Resistance and the Environment – Sources, Persistence, Transmission and Risk Management
CG: carbapenemase gene
CPE: carbapenemase-producing *Enterobacterales*
CPERL: CPE National Reference Laboratory Service
MDR: multidrug resistance
MGE: mobile genetic element
MIC: minimum inhibitory concentration
pMLST: plasmid multilocus sequence typing
16S-RMTases: 16S rRNA methyltransferases
ST: sequence type
WGS: whole-genome sequencing
