# Supplementary material for: Healthcare-related transmission of mobile genetic elements co-carrying blaNDM and 16S rRNA methyltransferase genes in multiple Enterobacterales
Source: Microb Genom. 2025 Aug 28;11(8):001473. doi: 10.1099/mgen.0.001473 (PMC12452189; doi:10.1099/mgen.0.001473)

Figure S1:Plasmid clusters identified by Pling. Each node represents a single plasmids and the edges are labelled with the containment distance and DCJ-indel distance.

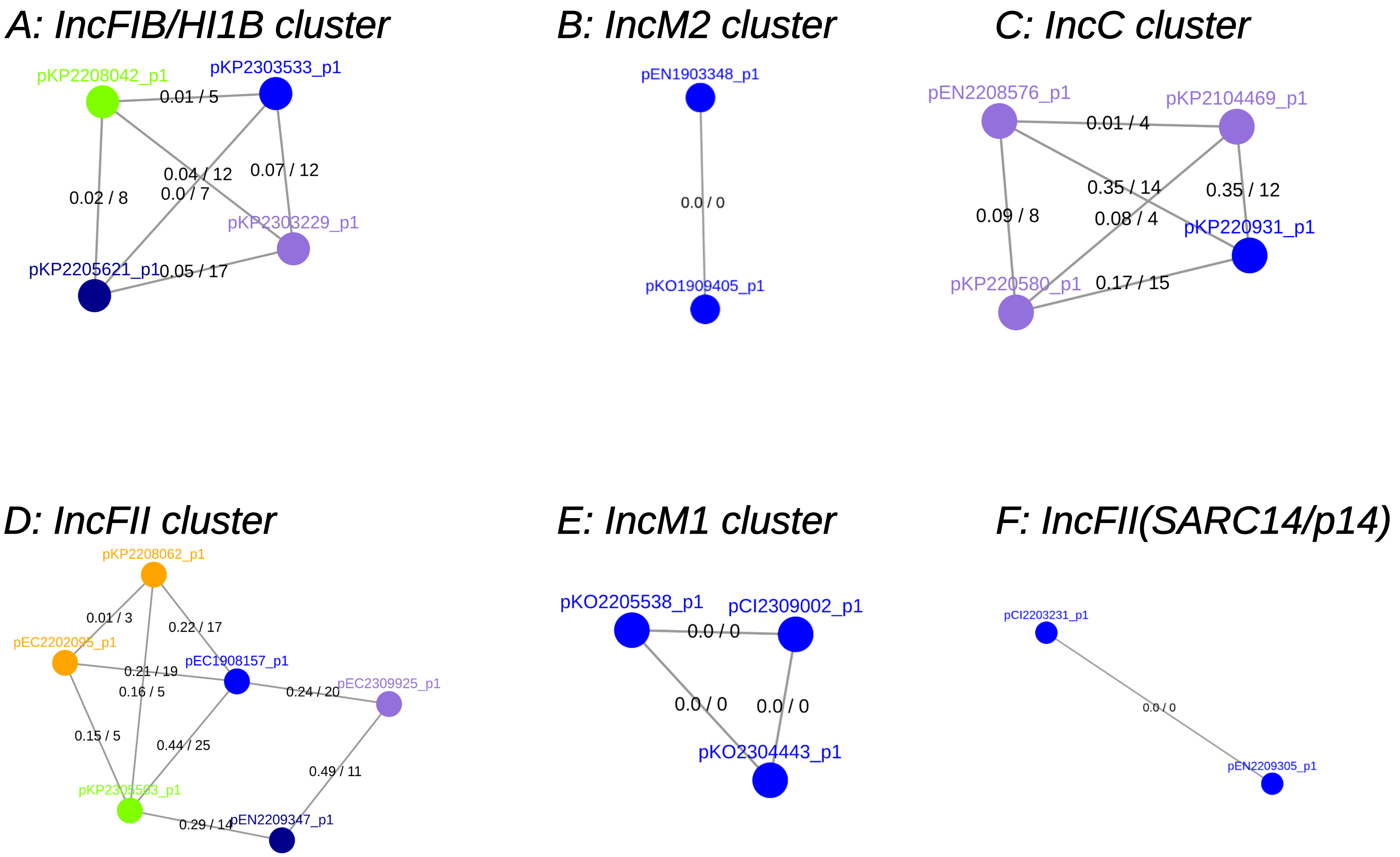

Supplement: Uncited Fig. S1. [file mgen-11-01473-s002.pdf]
